# Stiff-to-Soft Transition from Glass to 3D Hydrogel Substrates in Neuronal Cell Culture

**DOI:** 10.3390/mi12020165

**Published:** 2021-02-08

**Authors:** Gulden Akcay, Regina Luttge

**Affiliations:** Neuro-Nanoscale Engineering, Department of Mechanical Engineering and Institute of Complex Molecular Systems (ICMS), Eindhoven University of Technology, 5600 MB Eindhoven, The Netherlands; g.akcay@tue.nl

**Keywords:** hydrogel, 3D cell culture, brain-on-a-chip, SH-SY5Y cells, GelMA, PEGDA, mechanotransduction

## Abstract

Over the past decade, hydrogels have shown great potential for mimicking three- dimensional (3D) brain architectures in vitro due to their biocompatibility, biodegradability, and wide range of tunable mechanical properties. To better comprehend in vitro human brain models and the mechanotransduction processes, we generated a 3D hydrogel model by casting photo-polymerized gelatin methacryloyl (GelMA) in comparison to poly (ethylene glycol) diacrylate (PEGDA) atop of SH-SY5Y neuroblastoma cells seeded with 150,000 cells/cm^2^ according to our previous experience in a microliter-sized polydimethylsiloxane (PDMS) ring serving for confinement. 3D SH-SY5Y neuroblastoma cells in GelMA demonstrated an elongated, branched, and spreading morphology resembling neurons, while the cell survival in cast PEGDA was not supported. Confocal z-stack microscopy confirmed our hypothesis that stiff-to-soft material transitions promoted neuronal migration into the third dimension. Unfortunately, large cell aggregates were also observed. A subsequent cell seeding density study revealed a seeding cell density above 10,000 cells/cm^2^ started the formation of cell aggregates, and below 1500 cells/cm^2^ cells still appeared as single cells on day 6. These results allowed us to conclude that the optimum cell seeding density might be between 1500 and 5000 cells/cm^2^. This type of hydrogel construct is suitable to design a more advanced layered mechanotransduction model toward 3D microfluidic brain-on-a-chip applications.

## 1. Introduction

In the last decade, hydrogels have become increasingly important in emulating three-dimensional (3D) brain architecture in vitro. This class of polymeric materials provides biocompatibility, biodegradability, malleable mechanical properties, and a porous structure that allows the mass transfer of nutrients and cellular waste products by diffusion [1]. To this end, we built on our previous work using Matrigel as a 3D cell culture scaffold in so-called microbioreactor constructs on microelectrode array (MEA) chips [2] and in special dynamically modulating environments as demonstrated by a polydimethylsiloxane (PDMS) membrane-based actuator chip [3].

**Figure 1:**
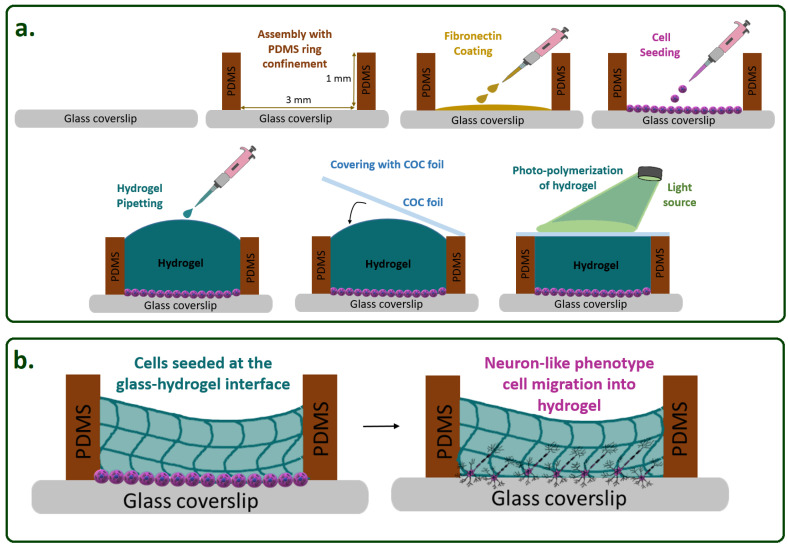
Schematic representation of cells seeded at a stiff-to-soft material interface. (**a**) Preparation of the 3D culture construct utilizing photo-polymerized hydrogels. (**b**) Before the differentiation and migration process right after cell seeding and subsequently sometime after, forming neuron-like phenotype cells and initiating neuronal processes. Polydimethylsiloxane (PDMS) and cyclic olefin copolymer (COC).

In this paper, we report on the effect of gelatin methacryloyl (GelMA) and poly (ethylene glycol) diacrylate (PEGDA) on SH-SY5Y cells in 3D. Figure 1 schematically depicts the concept of our rationale aiming to construct a model in which cells are sandwiched in between a stiff and a soft material interface. Observing this stiff-to-soft transition here from glass to a 3D hydrogel substrate in neuronal cell culture is the first step toward a new in vitro brain model.

To enable the observation of mechanotransduction processes in future biomedical studies applying this novel in vitro brain model, we introduce here a conceptual microfabricated device, starting from the well-known example of a 2D culture on tissue-treated glass plates and extending these cultures to 3D by exploring two different types of commercially available hydrogel systems in our SH-SY5Y culture experiments serving as a preliminary neural cell culture model. The selection of a suitable hydrogel system is crucial in the device design.

In more detail, utilizing hydrogels for this application can be defined into two categories, including natural hydrogels, such as gelatin, alginate, hyaluronic acid, fibrin, collagen, and synthetic hydrogels. Synthetic hydrogels include poly (ethylene glycol) (PEG), poly (acrylamide) (PAAm), and poly (caprolactone) (PCL), and poly (ethylene glycol) diacrylate (PEGDA). In addition, so-called semi-synthetic hydrogels, like gelatin-derived hydrogels, such as gelatin methacryloyl (GelMA) [4], and hyaluronic acid-based hydrogels, like HyStem™ and methacrylated hyaluronic acid (MeHA), are also well-established biomaterials [5,6]. Many different methods of patterning hydrogels are currently being investigated for their advances in 3D tissue engineering [7].

Considering the sensitivity of brain cells to the mechanical properties of their microenvironment 3D microfluidic brain-on-a-chip applications must take this factor into account [8]. In addition to having an appropriate stiffness of the hydrogel suitable for mimicking brain tissue’s constancy, the selected hydrogel for such models should also allow human induced pluripotent stem-cell (hiPSC)-derived neural progenitors to differentiate and migrate so to be able to define models at the neurocircuitry level in more detail [9,10].

Among the various hydrogels, PEGDA and GelMA have proven themselves for 3D tissue engineering applications in terms of their biocompatibility, biodegradability, and low cost [11]. PEGDA is a PEG derivative hydrogel that is fabricated through substituting the terminal hydroxyl groups of PEG with acrylates. PEGDA has two acrylate groups at each end of the PEG backbone [6]. Even though unmodified PEGDA has a lack of adhesion peptides, insertion of the acrylate side groups allows it to gain bio-functionality for encouraging tissue regeneration [12]. Gelatin is a polymer that can be obtained from the denaturation of collagen. It maintains natural cell-adhesive binding peptides like Arg-Gly-Asp (RGD) in the innate structure [13].

Given these beneficial properties of GelMA and PEGDA, we selected these materials for our investigations toward the design of a microenvironment. We envisaged that studying the migration behavior of SH-SY5Y neuronal model cells seeded in a sandwiched manner between a glass and a hydrogel substrate would also further elicit mechanotransduction processes in 3D microfluidic brain-on-a-chip applications.

## 2. Materials and Methods

### 2.1. Hydrogel Fabrication

#### 2.1.1. GelMA (Gelatin Methacryloyl) Preparation

A GelMA prepolymer solution was prepared with 5% (*w*/*v*) GelMA (900496, Sigma Aldrich, Saint Louis, MO, USA) in 0.01 mM Eosin Y (E6003, Sigma Aldrich), 0.1% (*w*/*v*) triethanolamine (TEA) (90279, Sigma Aldrich), 37 nM N-vinyl-2-pyrrolidinone (NVP) (95060, Sigma Aldrich), and phosphate buffered saline (PBS, LO BE02-017F, Westburg, Leusden, The Netherlands). The mixture was stirred on the hot plate at 65 °C and 300 rpm until completely dissolved. Next, the photo-polymerization of the hydrogel was performed by visible green light for 100 s at room temperature [14].

#### 2.1.2. PEGDA (Poly(ethylene glycol) diacrylate) Preparation

The PEGDA (455008, Sigma Aldrich, Saint Louis, MO, USA) hydrogel was performed by preparing two types of solution. The first was an Eosin Y solution was composed of Eosin Y disodium salt (E4382, Sigma Aldrich) 0.069% (*w*/*v*) and MiliQ water. The second solution, called the buffer solution, contained 100 mM NaCl, 10 mM 4-(2-hydroxyethyl)-1-piperazineethanesulfonic acid (HEPES), 1.5% (*v*/*v*) triethanolamine (TEA) (90279, Sigma Aldrich), and MiliQ water. After mixing 5% (*w*/*v*) PEGDA with buffer solution, added as a cross-linker 3.5 µL N-vinyl-2-pyrrolidinone (V3409, Sigma Aldrich) and then mixed by putting 10 µL Eosin Y solution. For the gelation process, PEGDA hydrogel mixture was photo-polymerized by exposing it to visible white light at room temperature for 10 min.

#### 2.1.3. Red-Colored Thermoset Gelatin Preparation

Red-colored thermoset gelatin (Dr. Oetker, strawberry jelly, The Netherlands) was prepared by mixing 100 g of powder and 400 mL boiling water. After being poured into the PDMS ring confinement, the gelatin was kept at +4 °C for 2 h to form a gel.

#### 2.1.4. Cell Culture and Differentiation

The SH-SY5Y cell line (94030304, Sigma Aldrich) was grown in T75 flasks Dulbecco’s Modified Eagle’s Medium: Nutrient Mixture F12 (DMEM/F12) (L0093, Biowest, Nuaille, France) supplemented with 10% Fetal Bovine Serum (FBS) (FBS; lot no. 11113, Bovogen, East Keilor [VIC], Australia) and 1% penicillin–streptomycin (LODE17-602E, Westburg, Leusden, The Netherlands) at 37 °C in 5% CO_2_. The cell medium was refreshed every two days and, the cells were maintained until they reached 80–90% confluence.

##### Hydrogel Construct Preparation

Upon reaching confluence, the cells were seeded in the PDMS ring confinement followed by adding a droplet of 10 µL of the hydrogel, which was flattened by placing a cyclic olefin copolymer (COC) foil on top during the photo-polymerization according to the scheme in Figure 1a.

##### Cell Loading

Before cell loading, the PDMS molds were sterilized with 70% ethanol for 5 min by being submerged. Subsequently, the ethanol was aspirated and dried in an incubator. Immediately after sterilization, the surface to be seeded with cells was coated with 20 µg/cm^2^ Fibronectin (FC010, Sigma Aldrich). On day 0, the cells were seeded in DMEM/F12 with the seeding medium replaced by differentiation medium#1, which was 10% FBS and 1% penicillin–streptomycin, and then the differentiation was performed. On day 1, differentiation medium#1 was prepared by adding 10 µM retinoic acid (RA) (RA; R2625, Sigma Aldrich) to DMEM/F12 with 10% FBS and 1% penicillin–streptomycin to induce neuronal differentiation. On day 3, differentiation medium#1 was replaced with differentiation medium#2, which contained 50 ng/mL brain-derived neurotrophic factor (BDNF) (B2795, Sigma Aldrich) plus DMEM/F12 with 10% FBS, 1% penicillin–streptomycin, and 10 µM RA to sustain the survival of the cell differentiation. Thereafter, the culture with GelMA was stopped on day 5 and prepared for fixation and further analysis, and, when we observed that the culture with PEGDA did not differentiate, it was discarded. The cultures for the cell seeding density experiment were stopped on day 6 and only analyzed by bright-field without fixation.

#### 2.1.5. Immunofluorescence Staining Analysis

After washing the cells with warm PBS three times for 5 min, the cells were fixed with 3.7% paraformaldehyde (104005, Merck Millipore, Darmstadt, Germany). For permeabilization of the cells, we used 1% Triton X-100 (Merck Millipore, Burlington, MA, USA) for 10 min at room temperature, followed by blocking in 10% normal goat serum (NGS, Thermofisher Scientific, Bleiswijk, The Netherlands) in PBS for 15 min at RT. The cells were incubated with the primary antibody in blocking solution (β-tubulin III 1:200, (Sigma Aldrich)) overnight at 4 °C. After three times for 5 min PBS washing steps, the cells were exposed to secondary antibodies (anti-mouse IgG (H + L) Alexa 647 1:200, Thermofisher Scientific, Bleiswijk, The Netherlands) and 2 drops/mL NucGreen® Dead 488 reagent (R37109, Thermo Fisher Scientific)) for 3 h at room temperature. Images were obtained with a Leica TCS SP5X confocal laser scanning microscope (Leica TCS SP5X, Leica, Milton Keynes, UK). The images were built up by using proper excitation wavelengths for the different applications.

## 3. Results

**Figure 2:**
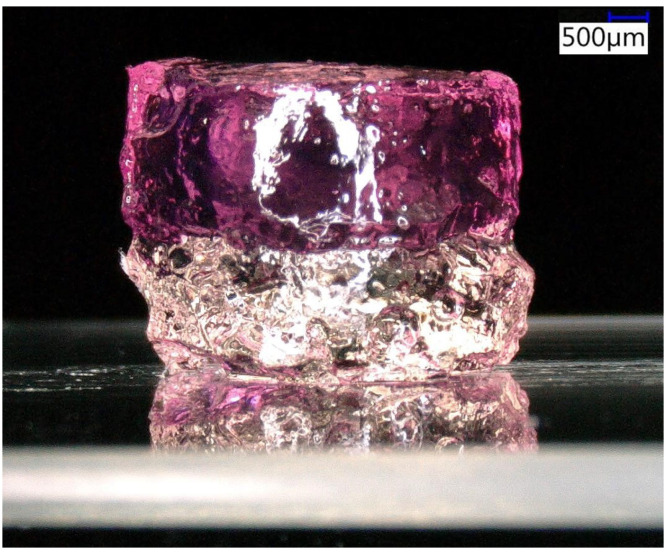
Concept of our stacked hydrogels cast on a standard microscope slide. Photo-polymerizable gelatin methacryloyl (GelMA) (transparent) on glass topped by a red-colored thermoset gelatin layer (red). The image was taken with a digital microscope (Keyence VHX-7000). The scale bar indicates 500 µm.

As a first proof-of-principle of the casting procedure illustrated in Figure 1, even with multiple interfaces, we created stiff-to-soft transition microenvironments by stacking photo-polymerized GelMA on glass followed by a red-colored thermoset gelatin layer as an example. The stacked hydrogels were kept in place during droplet dispensing using a simple, microliter-sized PDMS-ring for confinement on the glass substrate. Figure 2 depicts this layered construct.

**Figure 3:**
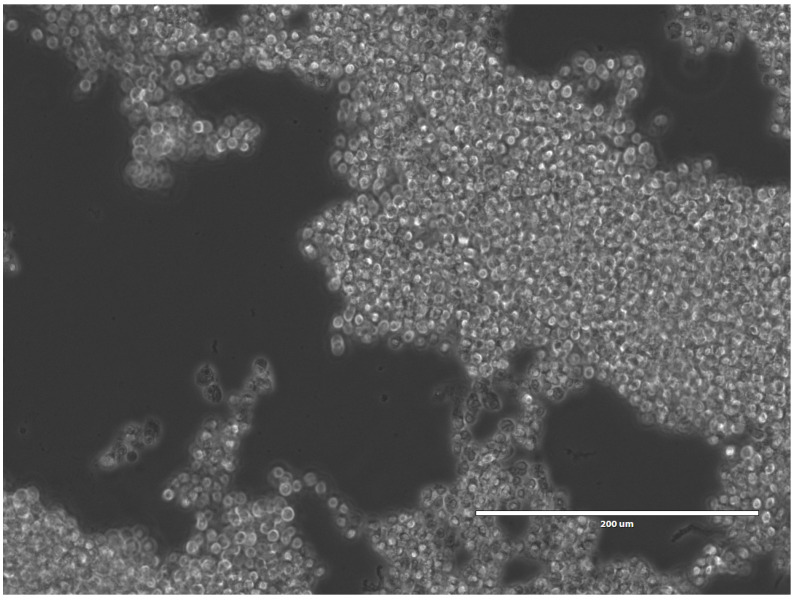
3D SH-SY5Y cells seeded in a PDMS ring confinement with a diameter of 3 mm on a microscope cover slip with poly (ethylene glycol) diacrylate (PEGDA) on top on day 1. The scale bar indicates 200 µm.

Consequently, we incorporated SH-SY5Y cells in this type of model by implementing either GelMA or PEGDA as the first layer atop of the cells and investigated the culture performance. We successfully cultured SH-SY5Y cells at 150,000 cell/cm^2^ seeding density in the cast GelMA construct, whereas PEGDA did not support the survival of the cells, which is indicated by the round shape of the cell bodies on day 1 (Figure 3).

In more detail, after the initial seeding of the cells in the PDMS ring confinement (3 mm diameter and 1 mm height) for both experiments, we cast hydrogel precursors and flattened their surfaces by placing a cyclic olefin copolymer (COC) foil on top during the polymerization step. The resulting height still varied greatly with this simple casting procedure and requires further optimization. However, this first trial for 3D SH-SY5Y cultures was performed according to our previous experience with 3D cultures of SH-SY5Y cells in microbioreactor cultures at 150,000 cells/cm^2^, and we differentiated the cells with RA to initiate neuronal differentiation on day 0 and added growth medium with BDNF on day 3. Then, the cultures were fixed on day 5 [15].

**Figure 4:**
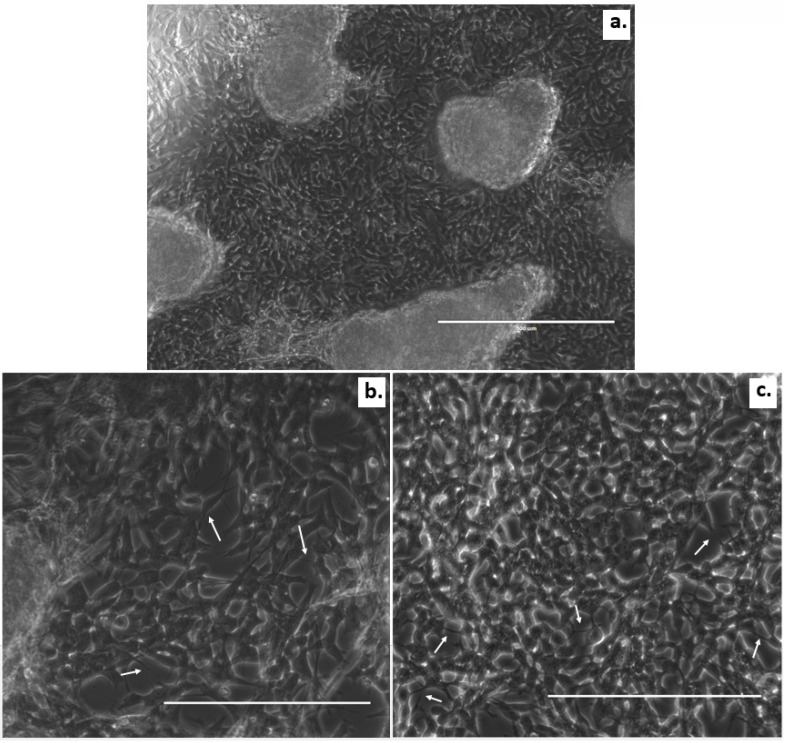
Morphology of the SH-SY5Y neuroblastoma cells in 3D culture. A larger field of view of the SH-SY5Y cells with GelMA on top on day 5 (**a**). The SH-SY5Y cells with GelMA on top imaged on day 1 (**b**) and day 5 (**c**). Images were taken using a EVOS FL microscope (Thermo Fisher Scientific, Eindhoven, The Netherlands) in the bright-field mode. White arrows indicate neuron-like phenotypes and neuronal outgrowths. The scale bars indicate 400 µm (a) and 200 µm (**b,c**).

Details of the fabrication and culture processes can be found in the Materials and Methods (Section 2). The bright-field images in Figure 4 show the SH-SY5Y cells inside the GelMA. The hydrogel supported cell attachment and survival as we anticipated, as GelMA has cell adhesion motifs, such as RGD. The 3D cultured SH-SY5Y neuroblastoma cells demonstrated an elongated, branched, and more spreading morphology resembling neurons. However, large cell clusters were also observed (Figure 4a). As highlighted in Figure 4b,c with white arrows, the GelMA hydrogel promoted neurite extension, the formation of a neuronal network, and cell communication.

**Figure 5:**
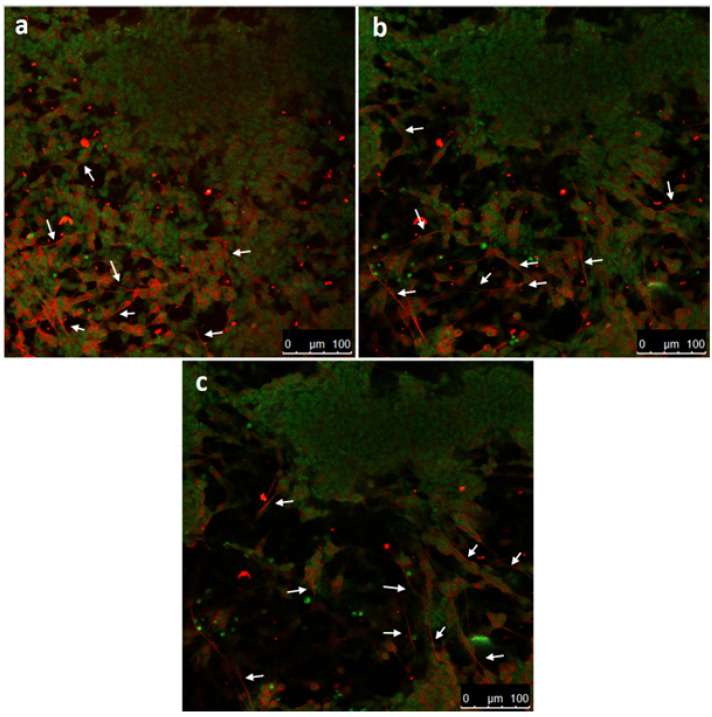
Confocal microscopy images of 3D SH-SY5Y cells in the GelMA. The staining displays neuron-specific protein β-Tubulin III (red) and cell nuclei dye (green). The maximum intensity projection of slices for the range of 0 to 60 µm from the bottom surface. The scale bars denote 100 µm. (**a**) Maximum intensity projection of slices for the range of 0 to 10 µm from the bottom surface. (**b**) Maximum intensity projection of slices for the range of 24 to 36 µm from the bottom surface. (**c**) Maximum intensity projection of slices for the range of 50 to 60 µm from the bottom surface. The slices were taken at an interval of 2 µm. Arrows in subfigures (a–c) indicate neuronal outgrowths.

We hypothesized that a stiff-to-soft material transition would promote neuronal migration. Based on this claim, neurons would migrate from glass, which is a very stiff material, to hydrogel, which is a noticeably soft material. To analyze the migration behavior of the neuronal cells, confocal microscopy z-stack images were examined. Figure 5 depicts the z-stacks of our culture results on day 5 with a starting cell density of 150,000 cells/cm^2^. In cultures with such a high seeding cell density, cluster forming is commonly reported, which we also observed with a larger field of view. The images here were taken between such clusters.

While cells in 3D culture conditions like ours can organize themselves across the volume; cluster forming will also depend on the total scaffold height available, and the migration speed of cells in the material will determine the final distribution of cells. As we can see in Figure 4, in addition to the accompanying neuron-like morphology, we observed that neural cells had, indeed, migrated into the GelMA hydrogel visible up to approximately 60 µm away from the bottom glass surface. Therefore, we can confirm a transition of SH-SY5Y cells from their 2D configuration on the glass into the 3D configuration of the GelMA rendering this biomaterial suitable for our intended application.

**Figure 6:**
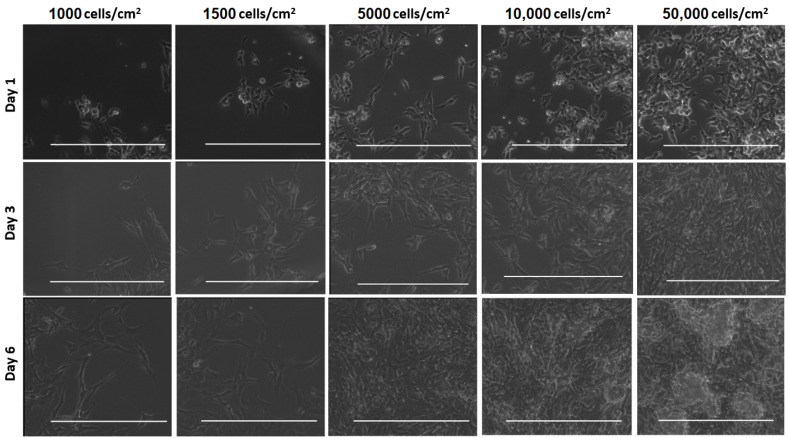
3D SH-SY5Y neuroblastoma cell cultures in GelMA within a PDMS ring on glass coverslips observed on day 1, 3, and 6, starting from different initial cell densities. Representative images were taken with an EVOS FL microscope in the bright-field mode. The scale bars denote 400 µm.

To provide a more extensive evaluation of our model regarding cluster forming, one-layer GelMA hydrogel constructs were established at various seeding densities. Subsequently, Figure 6 shows a series of bright-field optical microscopy images depicting differentiated cells embedded in GelMA. SH-SY5Y cells were plated according to the scheme in Figure 1 starting from different initial cell densities with 1000 cells/cm^2^, 1500 cells/cm^2^, 5000 cells/cm^2^, 10,000 cells/cm^2^, and 50,000 cells/cm^2^. Cells at all concentrations rapidly attached to the surface and proliferated over time. At low cell-seeding densities, such as 1000 cells/cm^2^ and 1500 cells/cm^2^, the cells remained visible as single cells up until day 6. Experiments with starting densities of 5000 and 10,000 cells/cm^2^ reached 70–80% confluency on day 3. The culture with a cell seeding density of 50,000 cells/cm^2^ was already nearly confluent on the first day. In the plated area that possessed the concentration of 50,000 cells/cm^2^, cellular aggregates began to appear on day 3 and became dominant on day 6. Large cell aggregates are not desired at this stage of cell culture as these cell clumps can lead to reduced diffusion of nutrients to and waste from the cells diminishing the cell viability and also hampering good control over the differentiation process by limiting the diffusion of the differentiation medium toward the core of clusters [16].

## 4. Discussion

The literature revealed positive effects of PEGDA hydrogel on several types of cells, such as cartilage cells [6] and pancreatic islet cells [17]. In our work here, we observed that SH-SY5Y cells were able to survive being embedded in GelMA but not in PEGDA (Figure 3). This could result from GelMA being a semi-synthetic hydrogel, which, therefore, permits taking advantage of the biological molecules inherent in the gelatin and enables control of its mechanical structure by modification with methacrylate side groups [18]. GelMA, being rich in cell-adhesive RGD peptides, specifically enhances the cellular attachment of neural cells and profoundly promotes neuro-regeneration, neural cell survival, migration, and differentiation when proper culture conditions are selected using this material as a scaffold [9,10,19].

To assess the differentiation of SH-SY5Y cells on the GelMA hydrogel, the cells were stained with β-tubulin III and NucGreen®. Despite β-tubulin III being an established neuronal cell marker, extending this assessment by microtubule-associated protein 2 (MAP2) and Tau protein (TAU) staining would provide valuable information regarding the cells’ states of differentiation [20,21,22,23,24,25]. To determine the migration, z-stacks were generated from 0 to 60 µm. In addition, SH-SY5Y cells can indeed proliferate and differentiate in the GelMA optical z-stacks at three different height ranges, which also confirmed that the cell outgrowths extended between different slices (Figure 5, see white arrows). In summary, photo-polymerized GelMA prepared in a microliter-sized confined layer demonstrated good cell proliferation, differentiation, and survival for SH-SY5Y cells against PEGDA. GelMA showed the transitioning of SH-SY5Y cells from the stiff glass substrate into the 3D microenvironment of the hydrogel. Previously, we similarly demonstrated SH-SY5Y in nanogroove-enhanced hydrogel scaffolds for a 3D neuronal cell culture as an easy access brain-on-a-chip model utilizing Matrigel instead [15]. Matrigel is a well-known hydrogel of a natural source implemented in many different types of tissue engineering applications requiring a 3D scaffold construct [26]. As Matrigel has rich protein components, such as collagen, heparan sulfate proteoglycan, and laminin, it is very good at mimicking the extracellular matrix (ECM). Therefore, Matrigel is one of the most established biomaterials. In addition to its use in neural tissue applications [27,28], Matrigel is broadly used in many other applications, such as the formation of adipose tissue [29] and cardiac muscle fibers [30], engineered liver organoids [31], and angiogenesis [27]. Therefore, it is reasonable to assume that, when we use Matrigel instead of the thermoset gelatin layer in the further refinement of our mechanotransduction model to two layers, the neural networks will also extend across the second boundary.

Modeling of native brain tissue in vitro requires a proper cell density to function as neurons in a 3D environment. We tested SH-SY5Y cells on GelMA hydrogel with different initial cell densities to acquire the most competent result (Figure 6). When using a low initial cell seeding density, the intercellular cell signaling will be increased due to interactions among the cells. The increase of the initial cell density continues until an ideal cell seeding density is achieved for a definite cell–ECM construction. On the other hand, once cultures are started with a higher initial cell seeding density, the cells suffer from large cell aggregations. Aggregate formation leads to inhomogeneity, inhibition of intercellular communication, and insufficient nutrient transport [32,33,34]. Especially for long-term cell cultures, the initial cell density is important to prevent the formation of large cell clusters. Thus, we noticed that the optimal initial cell density was between 1500 and 5000 cells/cm^2^.

Before we expand our model, we aim to reach a better understanding of the stiff-to-soft cell transition process. Figure 1 schematically depicts the preparation of the experimental steps, from cell seeding to casting the hydrogel (Figure 1a) and the initiation of the differentiation and migration process (Figure 1b). Potentially, we also expect that when differentiated neurons reach a new boundary with their axonal outgrowth, such as in the transition from a hydrogel 1 (here, GelMA) to a hydrogel 2 (not shown, e.g., Matrigel) with Young’s moduli E1 > E2, there is a high likelihood that neuronal somas can be enabled to be pulled up into this softer hydrogel region atop of the layer of GelMA by the activation of migration processes in a layered construct similarly to the work by Lozano R. et al., who utilized 3D printing to stack different types of hydrogels in their migration model [35,36].

GelMA has intrinsically high RGD cell adhesion peptides, which supports migration compared to non-enriched scaffolds, such as PEGDA, which is also often used as a biomaterial. High RGD components may also add to the porosity of the matrix, which may favor active cell displacement in such a construct. In our previous work, SH-SY5Y cells were also shown to migrate into Matrigel at approximately 80 µm from the bottom surface [15].

Considering the hypothesis formulated in the field of neuroscience that the migration of cells in the developing brain is observed from stiffer to the softer regions of tissue [37], one can also argue that such regions correlate with high/low concentrations of RGDs; with the result that, once stem cells have arrived in a region of lower RGD levels, the cells will stop migrating and begin to transition into a fully differentiated state. Therefore, an in vitro model that allows us to investigate controlled variation of such types of mechanotransduction parameters in detail could help to elucidate this hypothesis.

## 5. Conclusions

We demonstrated photo-polymerized GelMA in microliter-sized constructs on glass activated the 2D to 3D transition of differentiated SH-SY5Y cells in culture. Although starting from a very high cell seeding density, the cells survived and differentiated in GelMA, as confirmed by neuronal outgrowths. Immunofluorescence staining and confocal microscopy images displayed that cells migrated into the 3D microenvironment approximately 60 µm away from the bottom glass surface.

In this study, we also investigated the effects of the SH-SY5Y cell seeding density on GelMA hydrogels in 3D. To the best of our knowledge, lower concentrations of cell seeding were favored for the creation of a more convenient 3D microenvironment, facilitating the penetration of nutrients, water, and growth factors to all cells homogeneously, enabling intercellular communication and avoiding unwanted cell accumulation. Based on this experience, a cell seeding concentration between 1500 and 5000 cells/cm^2^ is sufficient in terms of neuronal cell survival, differentiation, the formation of neuronal outgrowths, and to create a 3D neuronal network in a hydrogel.

In conclusion, these results allow us to propose this type of a micro-casted hydrogel construct in designing a more advanced layered mechanotransduction model toward 3D microfluidic brain-on-a-chip applications.

## Data Availability

Data sharing is not applicable to this article.

## References

[1] Chai Q., Jiao Y., Yu X. (2017). Hydrogels for biomedical applications: Their characteristics and the mechanisms behind them. Gels.

[2] Bastiaens A.J., Frimat J.P., van Nunen T., Schurink B., Homburg E.F., Luttge R. (2018). Advancing a MEMS-based 3D cell culture system for in vitro neuro-electrophysiological recordings. Front. Mech. Eng..

[3] Xie S., Gardeniers J., Luttge R. (2018). Nanoscale membrane actuator for in vitro mechano-stimuli responsive studies of neuronal cell networks on chip. J. Micromech. Microeng..

[4] Ye W., Li H., Yu K., Xie C., Wang P., Zheng Y., Zhang P., Xiu J., Yang Y., Zhang F. (2020). 3D printing of gelatin methacrylate-based nerve guidance conduits with multiple channels. Mater. Des..

[5] Park S., Park K.M. (2016). Engineered polymeric hydrogels for 3D tissue models. Polymers.

[6] Choi J.R., Yong K.W., Choi J.Y., Cowie A.C. (2019). Recent advances in photo-crosslinkable hydrogels for biomedical applications. BioTechniques.

[7] Tenje M., Cantoni F., Hernández A.M.P., Searle S.S., Johansson S., Barbe L., Antfolk M., Pohlit H. (2020). A practical guide to microfabrication and patterning of hydrogels for biomimetic cell culture scaffolds. Organs-on-a-Chip.

[8] Kim H.N., Choi N. (2019). Consideration of the Mechanical Properties of Hydrogels for Brain Tissue Engineering and Brain-on-a-chip. BioChip J..

[9] Tsou Y.H., Khoneisser J., Huang P.C., Xu X. (2016). Hydrogel as a bioactive material to regulate stem cell fate. Bioact. Mater..

[10] Iwashita M., Ohta H., Fujisawa T., Cho M., Ikeya M., Kidoaki S., Kosodo Y. (2019). Brain-stiffness-mimicking tilapia collagen gel promotes the induction of dorsal cortical neurons from human pluripotent stem cells. Sci. Rep..

[11] Mazza G., Al-Akkad W., Rombouts K., Pinzani M. (2018). Liver tissue engineering: From implantable tissue to whole organ engineering. Hepatol. Commun..

[12] Papavasiliou G., Sokic S., Turturro M. (2012). Synthetic PEG hydrogels as extracellular matrix mimics for tissue engineering applications. Biotechnology Molecular Studies and Novel Applications for Improved Quality of Human Life.

[13] Al Rifai N., Hasan A., Kobeissy F., Gazalah H., Charara J. Culture of PC12 neuronal cells in GelMA hydrogel for brain tissue engineering. Proceedings of the 2015 International Conference on Advances in Biomedical Engineering (ICABME).

[14] Yanagawa F., Sugiura S., Kanamori T. (2016). Hydrogel microfabrication technology toward three dimensional tissue engineering. Regen. Therapy.

[15] Bastiaens A., Xie S., Luttge R. (2019). Nanogroove-enhanced hydrogel scaffolds for 3D neuronal cell culture: An easy access brain-on-chip model. Micromachines.

[16] Kropp C., Massai D., Zweigerdt R. (2017). Progress and challenges in large-scale expansion of human pluripotent stem cells. Process Biochem..

[17] Marchioli G., Zellner L., Oliveira C., Engelse M., de Koning E., Mano J., Karperien, van Apeldoorn A., Moroni L. (2017). Layered PEGDA hydrogel for islet of Langerhans encapsulation and improvement of vascularization. J. Mater. Sci. Mater. Med..

[18] Pepelanova I., Kruppa K., Scheper T., Lavrentieva A. (2018). Gelatin-Methacryloyl (GelMA) hydrogels with defined degree of functionalization as a versatile toolkit for 3D cell culture and extrusion bioprinting. Bioengineering.

[19] Fan L., Liu C., Chen X., Zou Y., Zhou Z., Lin C., Tan G., Zhou L., Ning C., Wang Q. (2018). Directing induced pluripotent stem cell derived neural stem cell fate with a three-dimensional biomimetic hydrogel for spinal cord injury repair. ACS Appl. Mater. Interfaces.

[20] Dehmelt L., Halpain S. (2005). The MAP2/Tau family of microtubule-associated proteins. Genome Biol..

[21] Hromadkova L., Bezdekova D., Pala J., Schedin-Weiss S., Tjernberg L.O., Hoschl C., Ovsepian S.V. (2020). Brain-derived neurotropic factor (BDNF) promotes molecular polarization and differentiation of immature neuroblastoma cells into definitive neurons. Biochim. Biophys. Acta-(Bba)-Mol. Cell Res..

[22] Gunhanlar N., Shpak G., Van Der Kroeg M., Gouty-Colomer L., Munshi S., Lendemeijer B., Ghazvini M., Dupont C., Hoogendijk W., Gribnau J. (2018). A simplified protocol for differentiation of electrophysiologically mature neuronal networks from human induced pluripotent stem cells. Mol. Psychiatry.

[23] Satir T.M., Nazir F.H., Vizlin-Hodzic D., Hardselius E., Blennow K., Wray S., Zetterberg H., Agholme L., Bergström P. (2020). Accelerated neuronal and synaptic maturation by BrainPhys medium increases A*β* secretion and alters A*β* peptide ratios from iPSC-derived cortical neurons. Sci. Rep..

[24] Nazir F.H., Becker B., Brinkmalm A., Höglund K., Sandelius Å., Bergström P., Satir T.M., Öhrfelt A., Blennow K., Agholme L. (2018). Expression and secretion of synaptic proteins during stem cell differentiation to cortical neurons. Neurochem. Int..

[25] Hansen S.K., Stummann T.C., Borland H., Hasholt L.F., Tümer Z., Nielsen J.E., Rasmussen M.A., Nielsen T.T., Daechsel J.C., Fog K. (2016). Induced pluripotent stem cell-derived neurons for the study of spinocerebellar ataxia type 3. Stem Cell Res..

[26] Klotz B.J., Oosterhoff L.A., Utomo L., Lim K.S., Vallmajo-Martin Q., Clevers H., Woodfield T.B., Rosenberg A.J., Malda J., Ehrbar M. (2019). A versatile biosynthetic hydrogel platform for engineering of tissue analogues. Adv. Healthc. Mater..

[27] Uwamori H., Higuchi T., Arai K., Sudo R. (2017). Integration of neurogenesis and angiogenesis models for constructing a neurovascular tissue. Sci. Rep..

[28] Lee S.W., Lee H.J., Hwang H.S., Ko K., Han D.W., Ko K. (2015). Optimization of Matrigel-based culture for expansion of neural stem cells. Anim. Cells Syst..

[29] Kawaguchi N., Toriyama K., Nicodemou-Lena E., Inou K., Torii S., Kitagawa Y. (1998). De novo adipogenesis in mice at the site of injection of basement membrane and basic fibroblast growth factor. Proc. Natl. Acad. Sci. USA.

[30] Bakunts K., Gillum N., Karabekian Z., Sarvazyan N. (2008). Formation of cardiac fibers in Matrigel matrix. Biotechniques.

[31] Garnier D., Li R., Delbos F., Fourrier A., Collet C., Guguen-Guillouzo C., Chesné C., Nguyen T.H. (2018). Expansion of human primary hepatocytes in vitro through their amplification as liver progenitors in a 3D organoid system. Sci. Rep..

[32] Li Q., Wang Q., Wang O., Shao K., Lin H., Lei Y. (2018). A simple and scalable hydrogel-based system for culturing protein-producing cells. PLoS ONE.

[33] Zhou H., Weir M.D., Xu H.H. (2011). Effect of cell seeding density on proliferation and osteodifferentiation of umbilical cord stem cells on calcium phosphate cement-fiber scaffold. Tissue Eng. Part A.

[34] Talukdar S., Nguyen Q.T., Chen A.C., Sah R.L., Kundu S.C. (2011). Effect of initial cell seeding density on 3D-engineered silk fibroin scaffolds for articular cartilage tissue engineering. Biomaterials.

[35] Tyler W.J. (2012). The mechanobiology of brain function. Nat. Rev. Neurosci..

[36] Lozano R., Stevens L., Thompson B.C., Gilmore K.J., Gorkin R., Stewart E.M., in het Panhuis M., Romero-Ortega M., Wallace G.G. (2015). 3D printing of layered brain-like structures using peptide modified gellan gum substrates. Biomaterials.

[37] Koser D.E., Thompson A.J., Foster S.K., Dwivedy A., Pillai E.K., Sheridan G.K., Svoboda H., Viana M., da F Costa L., Guck J. (2016). Mechanosensing is critical for axon growth in the developing brain. Nat. Neurosci..

